# Why Do “Digital Hamsters” Hoard but Never Consume? Configurational Pathways and Influencing Mechanisms of Digital Hoarding Behaviour Among Chinese Generation Z

**DOI:** 10.3390/bs15111575

**Published:** 2025-11-17

**Authors:** Chao Zhang, Jingwen Li, Yinze Hao

**Affiliations:** 1 School of Journalism and Communication, Henan University, No. 379, North Section of Mingli Road, Zhengzhou 450046, China; steven@henu.edu.cn (C.Z.); haoyinze@henu.edu.cn (Y.H.); 2 School of Psychology, Henan University, Jinming Campus of Henan University, North Section of Jinming Avenue, Kaifeng 475004, China; 3 Research Center for Oral Culture and Communication, Henan University, No. 379, North Section of Mingli Road, Zhengzhou 450046, China

**Keywords:** digital hoarding, digital behaviour, media dependency, Chinese Generation Z, fsQCA

## Abstract

The advancement of digital technologies has fostered distinctive behaviour patterns and cultural phenomena in online environments. As digital natives, Chinese Generation Z has gradually developed digital hoarding behaviour under the dual influence of technological convenience and emerging social pressures. Such behaviour functions as a coping mechanism for managing both real-world competitive anxieties and emotional uncertainties. Drawing on in-depth interviews with 35 Generation Z participants, this study employs fuzzy-set qualitative comparative analysis (fsQCA) to systematically examine the influence mechanisms of six antecedent conditions—media dependency, perceived usefulness, perceived ease of use, uncertainty avoidance, emotional attachment, and deletion barriers—on attitudes toward digital hoarding behaviour. The findings reveal two core configurational paths leading to high acceptance of digital hoarding: the first represents strategic knowledge accumulation pursued under the pressures of involution, where digital hoarding becomes a means of seeking competitive advantage; the second reflects a retreat into the digital sphere, where hoarding practices provide emotional security amid experiences of offline relational alienation. This study thus contributes a theoretical lens that moves beyond technological rationality toward social adaptation, explicating the intertwined emotional, psychological, and social drivers of digital hoarding. It also provides empirical insights for the design of supportive digital infrastructures and health education initiatives aimed at enhancing youth digital well-being.

## 1. Introduction

The evolution of media technologies has driven a shift in digital devices from purely instrumental tools to embodied cognitive extensions (Peng, 2022). The everyday integration of intelligent media has reconstructed the underlying logic of social cognition, giving rise to new forms of social networks based on data flows and diverse cultural patterns of human–machine symbiosis (S. Chen, 2022). With the aid of cloud storage and artificial intelligence technologies, individuals have transcended the physical limits of biological memory. From smartphone photo albums and social media bookmarks to layered archives in cloud drives and browser tabs, users continuously build vast personal digital repositories. The tendency to obsessively collect various types of digital content while rarely deleting them has become a common habit for many so-called “digital hamsters”. Among younger populations, this tendency toward digital hoarding has become in (Cao, 2023) creasingly pervasive (Zaremohzzabieh et al., 2024), emerging as one of the most salient generational traits of digital life. Consequently, many young users are caught in a repetitive cycle of constant acquisition and accumulation of massive amounts of digital content (Sedera et al., 2022).

However, explanations rooted solely in technological affordances or media-use perspectives fall short of capturing the underlying drivers of digital hoarding. According to the World Health Organization (WHO), suicide is the third leading cause of death among people aged 15–29 (World Health Organization, 2025). Excessive use of social media under conditions of information overload exacerbates social comparison and fear of missing out (FOMO), thereby amplify anxiety, self-devaluation, and diminished belonging, significantly increasing the risk of suicidal ideation (Tan et al., 2025; X. Wang, 2017). The Report on the Development of China’s National Mental Health (2023–2024) further shows that members of Chinese Generation Z—particularly those aged 18–24—exhibit significantly higher detected rates of depression, anxiety, and other forms of psychological distress compared to other age cohorts (Z. Chen, 2025). These difficulties, intertwined with insufficient social support and overreliance on smartphones, may trigger suicidal ideation (Huang et al., 2022). Within this generational context, Chinese Generation Z—born between 1996 and 2010—can be regarded as the “coevals” of the country’s internet development and digital transformation (Cai & Liu, 2023). Growing up in an environment saturated with smartphones, social media, and digital platforms, they exhibit a high degree of media adaptability and dependency. Compared with other cohorts, Generation Z demonstrates greater operational fluency with intelligent media and stronger psychological reliance on constant connectivity, becoming the first generation to mature entirely within an “always-online” environment. Their behavioural patterns often reflect digital prioritisation and habitual connectivity, in which platform algorithms, fragmented information, and the permanence of digital memory collectively foster overconsumption and passive exposure to online content. As a result, collecting and hoarding digital materials becomes a way to regain a sense of control and security in the face of informational and emotional overload. At the same time, Chinese Generation Z faces the convergence of educational, employment, and social pressures. Their digital hoarding behaviour can thus be interpreted as a psychological shift—transforming real-world stress into digital comfort. Cognitive schemas such as “saving brings reassurance” and “collection equals control” resonate strongly within this group, revealing that digital hoarding extends beyond technological choice to function as a compensatory mechanism for coping with social anxiety and survival stress.

Therefore, this study does not merely focus on Generation Z’s identity as “digital natives” but rather on the complex psychological mechanisms that underlie their digital behaviours. As sensitive adopters of emerging media technologies, their digital hoarding embodies multiple driving forces—technological acceptance, emotional attachment, and uncertainty avoidance. To reveal the pathways and influencing mechanisms behind this behaviour, the study extends the Technology Acceptance Model (TAM) through in-depth interviews and fuzzy-set qualitative comparative analysis (fsQCA), offering a configurational perspective for understanding new forms of informational behaviour in the digital media era.

## 2. Literature Review

### 2.1. Review of Research on Digital Hoarding Behaviour

The concept of digital hoarding originates from the psychological construct of hoarding disorder, which refers to a persistent difficulty in discarding possessions, resulting in excessive accumulation and associated distress (“Hoarding disorder”). This disorder involves individuals who continuously acquire and retain items, leading to significant emotional discomfort (Renschler & Freeman, 2023). In 2015, psychiatrist Van Bennekom and colleagues formally introduced the term digital hoarding, defining it as the uncontrolled accumulation of digital files that leads to stress and disorganisation, and suggesting its classification as a subcategory of hoarding disorder (van Bennekom et al., 2015). Research indicates that digital hoarding shares several features with physical hoarding, including excessive accumulation, difficulty discarding, and emotional distress (Sweeten et al., 2018). However, while physical hoarding is often associated with compulsive behaviour, digital hoarding tends to involve a more intentional acquisitive tendency and is generally regarded as a widespread behavioural pattern rather than a clinical disorder (X. Wu & Li, 2021). Its defining characteristics include content storage without thematic coherence, unstructured accumulation during the acquisition process, more severe tendencies toward excessive saving, highly disorganised and infrequently used data, difficulty in deletion, and strong emotional attachment to stored items (Jia et al., 2022).

Existing studies on digital hoarding behaviour primarily focus on dimensions such as excessive acquisition (Işleyen, 2019), inability to stop acquiring content (Bozacıa & Gökdeniza, 2020), disorganisation (Bozacıa & Gökdeniza, 2020), and difficulty discarding (Oravec, 2018). These dimensions encompass behaviours such as over-acquisition of digital content (Schüll, 2018), unconscious accumulation of digital resources (Sedera et al., 2022), disorganised storage of digital information, and obstructed deletion of digital content (Vinoi et al., 2024). These studies offer multiple perspectives for understanding digital hoarding. In China, academic research on this phenomenon has emerged relatively later than in the international arena, with a primary focus on contexts such as social media platforms and personal information management. For instance, the rapid growth of social media data combined with decreasing storage costs has resulted in widespread “storage without use” among university students, whose motivations for saving content often stem from psychological reassurance rather than practical necessity (L. Wang et al., 2022). Continuous information acquisition by individuals or organisations on social media platforms can lead to the persistent accumulation of digital information, often in disorganised forms with low utilisation rates (Zhu & Jiang, 2024). However, most domestic research to date has been qualitative and platform-focused (K. Wu & Zhou, 2025), with limited efforts toward constructing theoretical models or exploring configurational mechanisms (Dai et al., 2024). Consequently, systematic and dialectical examinations of digital hoarding as a collective behavioural phenomenon remain lacking in the Chinese scholarly literature.

### 2.2. Research on the Influencing Factors of Willingness to Engage in Digital Hoarding Behaviour

The willingness to engage in digital hoarding can be understood as an extended outcome of the Technology Acceptance Model (TAM), in which technological affordances serve as the premise, while psychological regulation and emotional attachment jointly drive the emergence and persistence of hoarding behaviours. Proposed by Davis (1989), the TAM identifies perceived usefulness and perceived ease of use as the two core antecedents that shape users’ acceptance of new technologies (Davis, 1989). Originally developed within a Western context characterised by technological rationality, the TAM emphasises instrumental judgments and efficiency optimisation. However, within the Chinese sociocultural environment, Generation Z’s media practices are deeply embedded in the structure of digital society. Unlike in Western settings, where technology is often treated as a neutral tool, Chinese youth—shaped by an intertwined context of intense digitalisation and collective competitiveness—regard technological use as a means of social participation and self-construction. Accordingly, TAM in the Chinese context extends beyond explaining whether individuals adopt technologies to a social–psychological framework that elucidates how individuals utilise technologies to manage social pressures, achieve psychological regulation, and construct digital identity. Currently, digital hoarding behaviour has yet to form a systematic theoretical framework. However, scholars have begun to reveal some underlying reasons for the persistent retention of digital content from technological, psychological, and emotional perspectives (Sweeten et al., 2018). From a technological perspective, the widespread adoption of mobile devices, the shift from physical to digital storage, and the significant reduction in storage costs have greatly facilitated users’ digital hoarding (L. Wang et al., 2022). Digital tools allow effortless preservation of large quantities of multimedia content—photos, videos, and documents—without storage constraints and with instant accessibility. Their efficiency, convenience, and negligible cost constitute key motivators (Sedera et al., 2022). Multiple qualitative studies have highlighted that technological contexts (e.g., expanded storage space, reduced costs) (Jia et al., 2023) and technological empowerment (e.g., automatic backup functions) influence this behaviour (L. Wang et al., 2022). Quantitative research further confirms that technological factors significantly impact digital hoarding behaviour (Z. Zhang & He, 2023). TAM posits that perceived usefulness and perceived ease of use constitute the core antecedents of users’ adoption of digital behaviours (Davis, 1989), and the convenience of the technological environment serves as a crucial basis for Chinese Generation Z’s willingness to hoard digital content. Yet, the classic TAM logic alone is insufficient to explain the persistence and irrationality of hoarding behaviour, necessitating the integration of psychological and emotional variables.

From a psychological perspective, media dependency theory provides an essential supplement to the TAM framework. It posits that as individuals’ reliance on media systems deepens, their cognition and behaviour become increasingly influenced by these media (DeFleur, 1990). Such dependency enhances users’ perceived usefulness and perceived ease of use of media technologies, thereby reinforcing their intention to continue using them (Carillo et al., 2017). For the Chinese digital-native Generation Z, this dependency extends beyond information acquisition to a profound functional trust and psychological reliance. In existing empirical research, intolerance of uncertainty has been identified as a well-established psychological construct, typically measured through items reflecting anxiety and stress caused by uncertainty (Freeston et al., 1994) and behavioural inhibition in the face of uncertain situations (Buhr & Dugas, 2002). Uncertainty avoidance plays a critical role in shaping users’ digital hoarding tendencies (L. Wang et al., 2022). The vast amount of information on social media platforms is often coupled with uncertainty regarding the individual value of the content. Consequently, users frequently adopt strategies such as loss aversion or risk aversion to avoid potential losses and negative outcomes (Z. Zhang & Liu, 2024). The inherent uncertainty of social media information conflicts with users’ intolerance of uncertainty, creating cognitive dissonance. To restore cognitive balance, users engage in digital hoarding as a means of seeking certainty (Qi, 2017). This forms a key psychological driver of digital hoarding. However, the basic TAM framework alone is insufficient to explain excessive preservation and irrational hoarding; thus, media dependency and uncertainty avoidance function as essential psychological supplements within the TAM-based explanatory structure.

From the emotional perspective, existing studies grounded in attachment theory have extended individuals’ attachment to physical objects to include digital content (Sedera et al., 2022). It has been found that compared to non-digital possessions, individuals who collect digital content experience a stronger emotional connection to the content, forming close bonds and deeper memories with the hoarded items (Vitale et al., 2018). For Chinese Generation Z, characterised by restrained emotional expression and a sense of relational alienation, digital platforms have become significant emotional storage spaces, enabling them to preserve memories and sustain interpersonal connections (Sweeten et al., 2018; Z. Wu & Ma, 2024). This attachment-based emotional structure transforms digital hoarding into a practice of emotional consolation and identity construction. Moreover, individuals with digital hoarding tendencies find it considerably more difficult to delete digital content than to discard physical possessions (Schiele & Hughes, 2013). Despite the prevalence of archival, cleanup, and smart classification tools, Chinese users often experience hesitation and procrastination—a psychological resistance to deletion—stemming from fears of losing part of their identity (Frost et al., 2011). Digital assets thus function as extensions of the self, symbolising personal identity (Cushing, 2013). For Chinese youth, deletion barriers arise from their expectation of the future value of stored content, emotional dependence, and the need to preserve identity symbols. These factors collectively provide a sense of security and control over daily life (T. Liu & Jia, 2023). In this sense, emotional attachment and deletion barriers should be regarded not as external to technology but as affective extensions of TAM—factors that shape users’ subjective perceptions of ease of use by increasing the psychological cost of deletion relative to the technical cost, thereby reinforcing the continuity and rationalisation of digital hoarding behaviour.

It is evident that the formation of digital hoarding behaviours is jointly driven by multidimensional factors. While the TAM highlights perceived usefulness and perceived ease of use to explain how technological affordances facilitate digital preservation, media dependency and uncertainty avoidance reveal adaptive mechanisms at the psychological level. Meanwhile, attachment theory and digital self-extension underscore the deeper emotional connections and identity-related motivations. The integration of these elements jointly constitutes the key drivers influencing Chinese Generation Z’s willingness to engage in digital hoarding.

### 2.3. Current Research

Existing studies have explored digital hoarding behaviour from multiple dimensions, constructing influence pathways involving technological convenience, psychological regulation mechanisms, and emotional attachment, thereby revealing the multifactorial causes of digital hoarding as a widespread behavioural tendency in digital environments. However, several limitations remain. First, research perspectives tend to be singular, often explaining behavioural causes from technical, psychological, or emotional dimensions in isolation, lacking holistic analyses of the interactive mechanisms among multiple factors. Digital hoarding behaviour typically intertwines functional dependence on tools, avoidance tendencies toward uncertain information, and emotional identification with digital content. Systematic studies that integrate these different mechanisms and identify their structural relationships are still lacking. Second, existing research lacks both cultural and theoretical depth in model construction. Most prior studies have adopted the TAM derived from Western contexts, emphasising the rational dimension of technology adoption while paying insufficient attention to its cultural adaptability in China. In fact, within a social environment characterised by the coexistence of collectivism and competitiveness, Chinese Generation Z’s digital hoarding behaviour demonstrates an extension from technological adoption to social adaptation. Therefore, incorporating variables such as media dependence, emotional attachment, and deletion barriers into the TAM framework to achieve a contextualised reinterpretation becomes a key pathway toward developing a localised theory of digital behaviour. Third, analytical methods have mainly relied on traditional statistical approaches such as linear regression, neglecting the complex features of behavioural causes, including multiple configurations and equifinality.

Therefore, this study extends the TAM by treating perceived usefulness and perceived ease of use as core technological variables, while integrating four additional psychological and emotional dimensions—media dependency, uncertainty avoidance, emotional attachment, and deletion barriers. Using the fsQCA approach, the study explores the configurational effects of multiple interacting factors to uncover the multi-path formation mechanisms of digital hoarding among Chinese Generation Z. This integrated framework aims to construct a culturally sensitive and nonlinearly explanatory model for understanding digital behaviour in the Chinese context.

### 2.4. Theoretical Framework and Research Questions

This study builds an analytical framework for digital hoarding behaviour among Chinese Generation Z based on the TAM. On the original TAM constructs of perceived usefulness and perceived ease of use, it further incorporates four expanded variables—media dependency, uncertainty avoidance, emotional attachment, and deletion difficulty—derived from the practical characteristics of digital hoarding and the literature review. The framework aims to analyse the formation mechanisms of digital hoarding in youth from the dimensions of technological cognition, psychological regulation, and emotional motivation. Methodologically, data were collected through in-depth interviews designed around six core variables. Expert scoring was applied to assign values to these variables, and fsQCA was employed to explore the multiple configurational relationships among them. The fsQCA method can reveal multiple pathways driving high acceptance of digital hoarding behaviour, overcoming the limitations of traditional linear analyses. By decoding the technological adaptation logic, psychological mechanisms, and cognitive biases underlying Generation Z’s digital hoarding behaviour, this study provides valuable insights for fostering healthy digital behaviour among Generation Alpha (individuals born after 2010) in the age of intelligent media (Ziatdinov & Cilliers, 2022). The findings also offer practical implications for enhancing young people’s digital literacy, psychological resilience, and healthy media engagement, aligning with China’s ongoing efforts to promote network literacy education and youth mental health development.

This study focuses on the digital hoarding behaviour of Chinese Generation Z, with an emphasis on the complex formation mechanisms of their acceptance attitudes. The research questions include the following: (1) How do psychological, technological, and media-related factors combine in different configurations to shape the acceptance of digital hoarding behaviour among Chinese Generation Z? (2) Among the multiple configurational pathways, are there representative and generalisable “typical paths”? What dominant cognitive logics underlying the high acceptance of digital hoarding behaviour do these paths reflect?

## 3. Methods

### 3.1. Research Design

Digital hoarding behaviour among groups is a complex phenomenon resulting from the interaction of multiple conditions across psychological, technological, and media dimensions. The relationships involved exhibit high complexity and diversity, necessitating more precise research methods to uncover the underlying mechanisms. Given that quantitative methods tend to overlook the nonlinear characteristics of such behaviour (Pan & Chen, 2021), this study employs fuzzy-set qualitative comparative analysis (fsQCA) to examine the acceptance attitudes toward digital hoarding behaviour among Chinese Generation Z. This method is suitable for variables without clear binary oppositions, allows for fuzzy membership scores, and avoids information loss caused by rigid calibration standards (G. Chen & Liu, 2024). fsQCA integrates quantitative and qualitative perspectives (H. Zhang et al., 2023), emphasising that the effects of conditions on outcomes are neither independent nor symmetrical (Gan et al., 2020). It can reveal the configurational combinations and causal pathways of complex social phenomena, providing an appropriate analytical approach to explore the influencing mechanisms and configurational patterns behind the acceptance of digital hoarding behaviour in this group.

### 3.2. Research Participants

This study focuses on Chinese Generation Z youth, a cohort that has grown up in a digital media environment and is highly familiar with the use of intelligent platforms. They serve as a representative group for examining digital behaviour and psychological mechanisms in contemporary society.

During participant selection, the researchers adopted a combined strategy of snowball sampling and purposive sampling. Recruitment information was disseminated through multiple channels, including university communities, youth organisations, online interest groups, and the researchers’ social networks. Participants were also encouraged to recommend other eligible individuals. To ensure heterogeneity and representativeness, the sample covered young people with diverse educational backgrounds, occupations, and media usage habits, including high school students, undergraduates, postgraduates, and professionals such as teachers, civil servants, doctors, media practitioners, and sales personnel. Inclusion criteria were as follows: (1) individuals aged between 15 and 29 years; (2) mainland Chinese residents or individuals who had continuously lived in China for more than six years; (3) regular users of digital devices with experience in saving or collecting digital content; and (4) voluntary participation with signed written informed consent. Exclusion criteria included (1) minors without parental consent; (2) individuals with severe psychological disorders, cognitive impairments, or an inability to complete the interview; and (3) incomplete or missing audio recordings or data.

In line with fsQCA guidelines, where the number of condition variables is fewer than seven, a sample size between 10 and 40 cases is recommended (Marx, 2010). Theoretical saturation was reached after the 32nd interview. To enhance representativeness and ensure data saturation (Braun & Clarke, 2006), three additional participants were interviewed, yielding a final sample of 35 individuals (18 male, 17 female).

This study fully acknowledges the potential selection bias introduced by the use of snowball and purposive sampling. To minimise the risk of overconcentration within the researchers’ social networks, several control strategies were implemented: (1) Multi-channel recruitment: In addition to the research team’s personal networks, anonymous recruitment posts were distributed through multiple platforms such as university counselling centres, youth entrepreneurship communities, and online forums to ensure sample diversity. (2) Stratified balance design: The research team continuously monitored sample composition during recruitment to maintain an approximately 1:1 gender ratio and to ensure representation across age groups and occupational categories (students, educators, medical staff, government employees, media workers, and freelancers). (3) Geographical distribution control: Participants were recruited from multiple regions, including Henan, Beijing, Jiangsu, Guangdong, and Sichuan, covering both first- and second-tier cities as well as some prefecture-level areas to reduce regional bias. (4) Heterogeneous referral principle: In the snowball sampling stage, participants were explicitly encouraged to refer individuals with different social attributes (e.g., gender, occupation, or educational background) to increase sample heterogeneity. Through these measures, the final sample achieved structural balance in terms of age, occupation, and geographic distribution, offering a comprehensive reflection of the diversity within Chinese Generation Z while substantially reducing the influence of selection bias on the study’s conclusions. All participants provided written informed consent after being fully briefed on the study’s purpose and procedures. The research team anonymised and encrypted all data for internal use only to ensure privacy and data security. Demographic information of the final 35 participants is presented in Table 1.

### 3.3. Variable Design

In qualitative comparative analysis (QCA), the selection of condition variables must follow the “super condition” principle—identifying a small number of core factors capable of explaining the comprehensiveness and regularity of case outcomes, thereby addressing the problem of limited diversity (Guo & Wang, 2021). Methods for selecting condition variables include the problem-oriented approach, research framework approach, theoretical perspective approach, literature induction approach, and phenomenon summarisation approach, which may be used complementarily (M. Zhang & Du, 2019).

**Figure 1:**
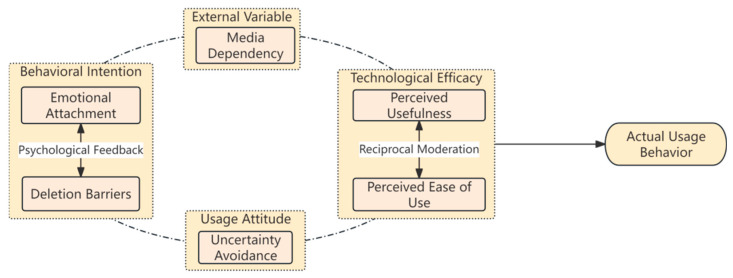
Attitudinal Model of Digital Hoarding Behaviour among Chinese Generation Z.

This study adopted a theoretical perspective approach and a literature induction approach to identify the conditional variables. From the theoretical perspective, the TAM was employed as the primary analytical framework for variable design, while contextual adaptations were made to reflect the sociocultural environment of Chinese Generation Z. From the literature induction perspective, existing studies were systematically reviewed in conjunction with the specific media practices of digital hoarding behaviour. On this basis, media dependency was introduced as an external variable influencing individuals’ choices and use of digital technologies. Perceived usefulness and perceived ease of use, as measures of technological efficacy, are treated as mutually influencing rather than following TAM’s original unidirectional assumption. This study emphasises their dynamic balance and reciprocal reinforcement. Behavioural intention is jointly constituted by emotional attachment and deletion barriers, which act as reciprocal psychological feedback loops, mutually reinforcing and generating the internal motivation for accepting digital hoarding behaviours. Uncertainty avoidance represents the usage attitude. These six variables interact to shape the multidimensional pathways through which Generation Z forms attitudes toward digital hoarding, as shown in Figure 1. The model retains TAM’s focus on the technological efficacy dimension while incorporating users’ emotional responses and cognitive regulation in digital media practices, aiming to present a multi-pathway influence structure of Generation Z’s attitudes toward the media technologies underpinning digital hoarding.

#### 3.3.1. Media Dependency

Media dependency can enhance users’ evaluations of a technology’s perceived usefulness and perceived ease of use. Accordingly, media dependency is positioned in this study as an external variable within the TAM framework (Carillo et al., 2017). Here, it refers to the sustained usage habits and functional trust that Chinese Generation Z develop toward media platforms (e.g., bookmarks, cloud storage, apps) when engaging in digital hoarding, representing an external condition shaping behavioural acceptance.

#### 3.3.2. Perceived Usefulness (PU)

Perceived usefulness (PU)—defined as the degree to which a technology is believed to enhance task performance—is a core determinant of technology adoption attitudes, motivations, and behaviours (Venkatesh et al., 2003). In digital hoarding, media systems have evolved from mere information intermediaries into instrumental platforms for knowledge accumulation, resource retention, and information retrieval. For Chinese Generation Z, PU refers to the belief that hoarding digital content enhances information security, resource access efficiency, and a sense of knowledge control, rendering it a “useful” strategic media practice. Importantly, this study conceptualises PU and perceived ease of use as mutually moderating: greater ease of use can improve usefulness evaluations, while high perceived usefulness can motivate skill acquisition, creating a positive feedback loop.

#### 3.3.3. Perceived Ease of Use (PEOU)

Perceived ease of use (PEOU) denotes the extent to which users perceive a technology as easy to operate (Davis, 1989). In this study, it refers to Chinese Generation Z’s familiarity and proficiency with the operational processes, saving functions, search capabilities, and classification mechanisms of digital tools used for hoarding. PEOU reflects whether hoarding is seen as a simple, effortless, and low-burden media operation. Together with PU, PEOU forms an interactive regulatory mechanism that jointly shapes acceptance levels and continuance intentions, influencing eventual behavioural outcomes.

#### 3.3.4. Uncertainty Avoidance

Usage attitude refers to the positive or negative feelings toward using a system and the evaluation of its consequences (Davis et al., 1989). Under platform-driven data architectures and storage logics, Generation Z often chooses digital hoarding as a safeguard against potential risks such as information loss, link expiration, or platform changes (Y. Liu et al., 2023). This tendency manifests in strategies of saving more, saving faster, and saving comprehensively. Uncertainty avoidance, as a cognitive-bias-type usage attitude, reflects defensive expectations that emerge when system instability is perceived, aiming to preempt threats through exhaustive saving behaviours (Hofstede, 1980). In this study, it refers to Chinese Generation Z’s inclination to adopt comprehensive storage, immediate bookmarking, and repeated backups when hoarding digitally, driven by a desire for information control and risk mitigation, thus reinforcing acceptance of the behaviour.

#### 3.3.5. Emotional Attachment

Emotional needs are a critical driver of digital hoarding among Chinese Generation Z and serve as an expression of behavioural intention. Once users develop psychological ownership over digital content, they form emotional attachments that link stored items to their self-concept (Chatterjee et al., 2013). The most immediate benefit of digital hoarding is instant access to stored material. Emotional attachment fosters this behaviour as a source of security and satisfaction, embedding affective value in the act of collecting. Here, emotional attachment denotes the deep emotional bonds and retention intentions that arise from emotional projection and identity association with stored content, acting as a key motivator for sustained engagement in digital hoarding.

#### 3.3.6. Deletion Barriers

Deletion barriers represent a widespread psychological resistance in Generation Z’s digital hoarding, manifesting as avoidance of data deletion and serving as a concrete behavioural intention (Vitale et al., 2018). In everyday media contexts, digital content becomes a repository of self-expression, experiential accumulation, and emotional anchoring. “Not deleting” thus emerges as a coping strategy to alleviate choice anxiety and feelings of losing informational control (Luxon et al., 2019). In this study, deletion barriers refer to Chinese Generation Z’s difficulty in making deletion decisions due to future uncertainty, emotional bonds, and cognitive load, fostering persistent resistance to deletion.

Together, media dependency, perceived usefulness, perceived ease of use, uncertainty avoidance, emotional attachment, and deletion barriers form the core condition variables spanning external dependency, technological efficacy, usage attitude, and behavioural intention. The attitude toward digital hoarding acceptance—the outcome variable in this study—captures the composite psychological orientation of individuals when saving, accumulating, and managing content via intelligent media. This orientation encompasses both cognitive evaluations of functionality, utility, and ease of use, and affective dispositions grounded in emotional bonds, risk avoidance, and habitual practices. It serves to assess whether, within current media structures and digital practices, Chinese Generation Z tends to recognise, support, and sustain patterns of digital hoarding, thereby revealing the configurational logic and generative pathways of their media-behaviour cognition.

### 3.4. Data Collection

This study employed in-depth interviews as the primary data collection method. This approach combines a structured framework with flexibility, ensuring comprehensive coverage of core themes while capturing rich, qualitative insights into participants’ personal experiences (Kallio et al., 2016). Prior to data collection, three researchers jointly developed the interview protocol, drawing on the research questions, literature review, and theoretical framework. A pilot study was conducted to identify any ambiguous or misleading questions (Kunselman, 2024). The design of the interview outline was grounded in the preceding literature review and theoretical framework, centring on six core variables: media dependency, perceived usefulness, perceived ease of use, uncertainty avoidance, emotional attachment, and deletion barriers. Six thematic modules were constructed accordingly, each containing two to three open-ended questions aimed at encouraging participants to freely articulate their authentic experiences related to each variable. For example, “In what ways do you usually save your digital content? Do you find these methods convenient?” (perceived ease of use); “Do you think saving these materials helps your future study or work?” (perceived usefulness); ”When you clean up or delete files, do you ever feel hesitant or worried?” (deletion barriers). The interview outline was refined through two rounds of expert review and two pilot interviews to ensure the neutrality and semantic clarity of question wording and to avoid any leading prompts.

After participants were fully informed of the research details—including the researchers’ identities, study objectives, and interview content—and had signed written consent forms, the interviews were conducted by the second and third authors. The second author (female) is an Assistant Research Fellow at the Center for Oral Culture Communication Studies, Henan University, with long-term research interests in media consumption behaviour and youth digital content management, and extensive experience in analysing the cognitive structures and behavioural pathways of digital hoarding among young people. The third author (male) is a master’s student at Henan University, focusing on digital media use and youth behaviour, with prior interviewing and data-processing experience. Interviews were conducted in Mandarin Chinese, either online or offline, depending on participants’ schedules and locations. Each session lasted approximately 50 to 100 min. With participants’ permission, all interviews were audio-recorded in full, accompanied by field notes and observational records, and subsequently transcribed, resulting in a total of approximately 487,000 words of textual data. To ensure confidentiality and data integrity, all materials were archived in encrypted devices with access strictly limited to the research team. Three researchers independently conducted multiple rounds of text reading and extracted representative statements and keywords corresponding to the six core variables. Guided primarily by a theory-driven inductive approach, this process identified recurring expressions and categorised participants’ responses into typical formulations of variable characteristics. For example, statements such as “Saving makes me feel at ease” or “I don’t dare delete anything” reflected a high level of emotional attachment; “I save materials for convenient review” or “These files will be useful in the future” indicated a high level of perceived usefulness; and “I often worry I’ll regret deleting something” or “I’m afraid I won’t find it later” represented uncertainty avoidance. These representative expressions were then discussed and consolidated into a set of semantic indicators for each variable, which subsequently served as the basis for expert scoring. This procedure established a logical transition from qualitative text analysis to quantifiable data.

### 3.5. Variable Assignment and Calibration

In the variable calibration stage, the research team invited five senior experts with experience in QCA research and backgrounds in digital media behaviour to independently score participants’ responses based on the established semantic indicator sets. A five-point intensity scale (1–5) was adopted instead of a binary classification. Scores of 4–5 were assigned when participants repeatedly and explicitly expressed attitudes consistent with a given variable or demonstrated strong tendencies; scores of 2–3 were used when attitudes were ambiguous or mentioned occasionally; and a score of 1 indicated no relevant expression or explicit denial. This scoring criterion captured the degree of variation in participants’ responses and avoided reducing complex psychological and behavioural tendencies to a simple binary judgment. Through this approach, the study achieved a systematic conversion from qualitative data to quantitative calibration.

To ensure fairness and consistency, before the formal scoring process, the research team organised an “expert calibration training session” to ensure inter-rater consistency. During the session, the experts were thoroughly briefed on the definitions of the six variables, the logic of the scoring procedure, and boundary cases in order to standardise the evaluation criteria and minimise subjective discrepancies. Based on the previously developed semantic indicator sets, experts rated participants’ responses using a five-point intensity scale (1–5) to reflect the strength of their attitudinal tendencies. During scoring, outlier treatment and standardisation methods were applied to reduce bias. For each variable, the mean score of responses to 2–3 relevant questions was calculated as the final score. After the scoring process, the research team calculated the inter-rater reliability to assess the consistency among experts’ evaluations. In this study, Krippendorff’s Alpha coefficient (α) was used, computed as follows:

$$\alpha =1-\frac{D_{O}}{D_{E}}$$

where D_O_ represents the observed disagreement, and D_E_ denotes the expected disagreement. When α ≥ 0.80, the coding results are generally considered to have high reliability (Krippendorff, 2018). The overall reliability coefficient in this study was α = 0.87, which is significantly higher than the conventional threshold of 0.80, indicating a high level of internal consistency among expert ratings. To verify the rigour and accuracy of the assignment process, eight participants were re-contacted and confirmed that the assigned values aligned with their actual attitudes and experiences. During the scoring and feedback process, some experts also provided constructive suggestions. For instance, one expert noted, “Although some participants did not explicitly express ‘concern about losing content,’ statements such as ‘I usually back up a copy’ can be regarded as an indirect indication of uncertainty avoidance”. In response, the research team supplemented the scoring guidelines to include indirect expressions as valid positive indicators, thereby enhancing the comprehensiveness and consistency of the coding. Another expert recommended that, when scoring perceived ease of use, both usage frequency and operational proficiency should be considered. Accordingly, the team adjusted the scoring reference standards to ensure that the quantified results more accurately reflect the intensity of participants’ behaviours.

For fsQCA, qualitative data must be transformed into a form suitable for set-theoretic analysis, with all variables calibrated into membership scores between 0 and 1. Following Ragin’s guidelines, three calibration anchors were defined for each variable: full membership (95th percentile), the crossover point (50th percentile), and full non-membership (5th percentile) (Ragin, 2009). The calibration anchors used in this study are presented in Table 2. After calibration, the dataset was processed using fsQCA 4.1 software for subsequent analysis.

## 4. Findings from fsQCA

### 4.1. Necessity Analysis of Single Variables

An important criterion for determining whether a condition is necessary is its consistency score. When the consistency of a single condition reaches or exceeds 0.90, the condition can be considered a necessary antecedent of the outcome variable. The calibrated data were imported into fsQCA 4.1, and a necessity test was conducted for all condition variables, yielding 12 results, as shown in Table 3.

Among all individual condition variables, the highest consistency value was 0.791599—none reached the 0.90 threshold. This indicates that none of the six condition variables has a significant causal relationship with the outcome on its own. Therefore, no single necessary condition exists, and further configurational effect analysis is warranted.

### 4.2. Configurational Effect Analysis

Configurational analysis examines how different combinations of condition variables jointly explain the outcome variable (Du et al., 2021). Given the sample size, the case frequency threshold in the fsQCA analysis was set to 1, ensuring that each configuration included at least one valid case. The consistency threshold was set to 0.8, guaranteeing that the identified configurations demonstrate a high degree of causal sufficiency (Ragin, 2009; Schneider & Wagemann, 2012) Combinations with a Proportional Reduction in Inconsistency (PRI) greater than 0.65 were coded as having the outcome 1, and those with PRI ≤ 0.65 were coded as not having the outcome 0 (Greckhamer, 2016). On this basis, the analysis generated complex, intermediate, and parsimonious solutions. The intermediate solution was chosen for further analysis, as it avoids the overly fine-grained causal distinctions of the complex solution and the underspecified mechanisms of the parsimonious solution. Following standard practice (Du & Jia, 2017), conditions present in both the intermediate and parsimonious solutions were identified as core conditions, while those appearing only in the intermediate solution were identified as peripheral conditions. The final configurational results are presented in Table 4.

As shown in Table 4, the consistency of each individual configuration and the overall solution exceeds 0.90—above the fsQCA requirement of 0.75 for all configurations (Ragin, 2006)—indicating strong explanatory power for high acceptance of digital hoarding among young people. The six configurations collectively have a coverage of 0.626777, meaning the model accounts for approximately 63% of cases, representing a relatively good model fit.

### 4.3. Robustness Check

To avoid spurious results, a robustness check was conducted by adjusting the consistency threshold from 0.80 to 0.85 and 0.90. When the consistency threshold was raised to 0.85, all six configurational paths remained stable, with no substantial changes in path structure or core conditions; the solution coverage slightly decreased to 0.6089, while consistency increased to 0.9256. Upon further increasing the threshold to 0.90, five configurational paths still met the requirement, whereas one marginal configuration was excluded due to slightly lower consistency. Under this condition, the solution coverage was 0.5932, and consistency rose to 0.9311. These results indicate that the paths of each configuration, as well as the solution coverage and consistency, exhibit no significant changes, demonstrating the robustness of the findings. The analysis conclusions are therefore reliable and carry practical reference value.

### 4.4. Conclusions from Configurational Analysis

Table 4 suggests that high acceptance of digital hoarding among Chinese Generation Z is not driven by any single factor but rather emerges from the interplay of multiple conditions. The mechanisms of these conditions vary across configurations:

#### 4.4.1. Configuration 1

This path can be summarised as “Instrumental Rational Type”, showing a digital hoarding logic oriented toward practical goals and risk prevention. Perceived usefulness and uncertainty avoidance act as core conditions, with media dependency providing peripheral support. Emotional attachment and deletion barriers have little effect, suggesting a lower affective need and minimal concern over deletion constraints. This configuration reflects a pragmatic strategy aimed at minimising risks of forgetting or information loss by creating a controlled memory safeguard.

#### 4.4.2. Configuration 2

This path reflects “Practical-Oriented Type”, where individuals are primarily driven by tool effectiveness and functional cognition. Perceived usefulness and uncertainty avoidance remain key drivers, supported secondarily by emotional attachment and deletion barriers. Perceived ease of use and media dependency play limited roles. Even when technical operations are challenging, individuals tend to retain content, indicating that utility outweighs usability or usage frequency in shaping acceptance.

#### 4.4.3. Configuration 3

This path can be defined as “Rational Minimalist Type”, emphasising goal-oriented and rational control of tool use. Perceived usefulness serves as the core driver, with perceived ease of use providing secondary support. Media dependency, uncertainty avoidance, and emotional attachment show no influence, and deletion barriers are absent as a core condition. This suggests that tool-oriented and task-driven motivations overshadow emotional or risk-avoidance considerations.

#### 4.4.4. Configuration 4

This path can be summarised as “Convenience–Defense Type”, highlighting the psychological logic of coexisting media operation convenience and risk prevention. Perceived ease of use and uncertainty avoidance are the primary drivers, underscoring the importance of accessible and user-friendly digital tools. Emotional attachment plays a minor role, while media dependency, perceived usefulness, and deletion barriers are less influential. This pathway reflects a preference for convenience and preparedness in the face of uncertainty.

#### 4.4.5. Configuration 5

This path can be defined as “Rational Efficacy Type”, characterised by trust in tools and multifunctional utility. This pathway emphasises functionality, convenience, and uncertainty avoidance, with deletion barriers playing a supporting role. Emotional attachment and media dependency have weaker effects. The pattern reflects high trust in, and reliance on, the multifunctionality of digital tools, with decisions grounded in rational efficiency rather than platform affinity or emotional triggers.

#### 4.4.6. Configuration 6

This path can be summarised as “Integrated Defense Type”, showing rational strategic features driven by multidimensional perceptual synergy. Functionality, convenience, and uncertainty avoidance are again central, supplemented by emotional and media dependency as peripheral supports. This configuration highlights a multidimensional sense of security derived from synergistic technical advantages, positioning digital hoarding as a rational strategy shaped by the convergence of favourable conditions rather than a single motivating factor.

## 5. Discussion

From the six configurational paths identified above, it can be observed that while the specific combinations of conditions shaping Chinese Generation Z’s willingness toward digital hoarding vary, they generally converge into two overarching tendencies: one is grounded in functional rationality, emphasising instrumental value and operational convenience; the other is guided by emotional attachment, highlighting psychological security and risk avoidance. Based on this distinction, the study further consolidates the six paths into two prototypical models, thereby providing a clearer understanding of the underlying logics driving Generation Z’s willingness to engage in digital hoarding, as shown in Table 5.

### 5.1. Utility-Integration-Driven: Functionality-Oriented Digital Hoarding Pathways

The utility-integration-driven pathway is anchored by perceived usefulness as the core condition, integrating perceived ease of use, uncertainty avoidance, and media dependency across multiple technical and psychological dimensions. This pathway reflects the interaction between Chinese Generation Z youth and the dual pressures of intense social competition and a deeply mediatised environment, forming a digital hoarding logic aimed at maximising efficacy and minimising risks. Rather than mere tool usage, it represents a cognitive safety net actively constructed by individuals to alleviate the anxiety caused by uncertainties in academic and career development.

The most distinctive feature here is the prioritisation of functional efficacy. In essence, it is a strategy to cope with the “liquid modernity” (Bauman, 2018) of contemporary society. The contradiction between the infinite supply of digital content and the finite nature of human cognition (Z. Zhang & Liu, 2024) becomes especially acute among Generation Z, who face harsh demands for constant “self-optimisation” within the “achievement society” (Han, 2019). Their bookmarking behaviour is a highly rational act of cognitive resource accumulation, aimed at enhancing their capacity for information allocation and resource management in fierce competition, thereby gaining marginal advantages. The 2025 Gen Z Workplace Outlook shows that more than half of Gen Z respondents report experiencing high-intensity stress in daily work and life (ManpowerGroup, 2025). Under such a high-pressure social environment, young users tend to hoard valuable digital content as reserves to cope with unknown challenges. Among the 35 interview participants, 26 explicitly stated that their saving behaviour was motivated by considerations of “potential future usefulness.” Of these, 12 out of 18 male participants and 9 out of 17 female participants mentioned that “hoarding” was related to occupational or academic anxiety. Most participants regarded the saving behaviour as a preparatory strategy, reflecting a clear orientation toward instrumental rationality. “Most of what I save is because I think ‘I might need it later,’ like study materials, lecture notes, or work templates. Having more preparation means less anxiety.” (F9) “My favorites folder is my second brain. I save things instantly when they come to mind—it’s convenient and reassuring.” (M2) To gain an advantage in competition, individuals must continuously learn and create new value to consolidate their positions (Lan, 2022). Digital hoarding thus emerges as a delayed-use but real-time-control information management strategy, a structural response to uncertainty in a complex digital environment driven by structural anxiety.

Chinese Generation Z youth within this pathway generally demonstrate high media operation proficiency and strong platform adaptability, reflecting both mastery and trust in media tools. They grew up in a “mobile phone childhood” (Zheng, 2025), with media platforms embedded as part of their developmental environment. As a result, they generally regard saving as a reflection of “self-efficiency management,” using platform features efficiently to build personal knowledge systems. Twenty participants reported that they regularly categorise and organise folders, “keeping materials as orderly as archives,” gradually treating media platforms as an “external brain” for managing their knowledge, helping them cope with the anxiety generated by societal time pressures (Neugarten et al., 1965). “I create different folders by theme, like study, travel, and food, each categorised for easy access later. This helps me quickly restore a sense of order when things feel chaotic.” (M5) “The search and recommendation functions on my phone are so strong that I can instantly find what I originally saved, without repeated filtering.” (F11) “Now cloud drives and note-syncing are so convenient. Even if I switch devices, everything stays synced—it gives me peace of mind.” (F17) Thus, media dependence in this pathway has gone beyond traditional notions of “attachment” and evolved into a media coordination mechanism, whereby young people actively integrate platform affordances with personal needs.

However, from a critical perspective, although the utility-integration-driven pathway exhibits high rationality and self-regulation, it also conceals an underlying overreliance on efficiency and control. In an environment where information updates rapidly, content may become invalid at any time and job–skill mismatches remain common (ManpowerGroup, 2025), Generation Z tends to adopt a “better too much than too little” approach to storing valuable information. This apparent rationality and order may, in fact, reflect an attachment rationalised by structural social anxiety. In the interviews, 18 participants admitted that “even though I know storing so much is unnecessary, I feel uneasy if I don’t,” indicating that rational strategies have partially transformed into emotional responses. “Once a link becomes invalid, you can’t find anything, so I just save it right away to avoid future regret. It’s like finding a job now—the requirements change so much, and without learning more skills you can’t keep up.” (F12) “I always sync downloaded materials to the cloud, so I don’t worry about losing my phone or deleting files by mistake.” (M11) Through comprehensive saving and repeated backups, they build a sense of informational security, functioning also as a psychological compensation mechanism against career uncertainty. When “storing” becomes a psychological comfort against an uncertain world rather than a tool-based behaviour grounded in real needs, rationality begins to transform into a latent burden. This implies that digital hoarding should not be simply classified as a functional behaviour or a pathological symptom; rather, it should be understood as a “marginal state” situated between rationality and compulsion.

The utility-integration-driven pathway reveals that digital hoarding among Chinese Gen Z youth emerges from the dual pressures of external social competition and internal career development anxiety, forming a cognitive survival strategy that combines strategic rationality with media adaptability. This pathway reflects the proactive adjustment and cognitive planning capacities of young people in media practices. In essence, in an era of social involution, they attempt to leverage media infrastructures to enhance their sense of control, constructing a cognitive order in the digital–intelligent environment—where content redundancy and choice anxiety coexist—based on the principle that “storing is managing, and collecting is mastering.” However, it also reveals the internal imbalance they experience in the process of over-rationalisation.

### 5.2. Emotional-Security–Attached: Emotionally Driven Digital Hoarding Pathways

The emotional-security–attached pathway (primarily represented by Pathways 2 and 4) highlights the non-rational drivers behind digital hoarding among Chinese Gen Z youth, specifically their tendency to use digital spaces as emotional outlets and safe havens in the context of interpersonal alienation, high-pressure social rhythms, and profound uncertainty about the future. This type of pathway represents a behavioural mode of digital escapism (Subudhi, 2020), with its core being the construction of a controllable and comforting private digital world through hoarding behaviours, thereby compensating for and buffering against the psychological pressures of the real world.

The core variable combination in this pathway is the synergistic presence of uncertainty avoidance and emotional attachment. On one hand, Chinese Gen Z are often considered a “lonely” generation: the aftereffects of the one-child policy and intense academic competition have made them more inclined to seek emotional connection and belonging online. At the same time, the anxieties of “difficult employment” and “involution” in Chinese society fuel their sense of insecurity about the future. Among the 35 participants, 21 explicitly reported that “deleting content causes anxiety or a sense of loss,” with a higher proportion among female respondents (12 out of 17). As such, their digital hoarding behaviour has gone beyond utilitarian purposes of knowledge retention, evolving instead into a psychological coping mechanism against feelings of loss of control: “Some of the things I’ve saved I rarely ever look at, but I just can’t delete them. Seeing them there gives me a lot more sense of security. In real life, many things slip away, but at least my digital world feels full and stable.” (F8) This indicates that digital hoarding has become a widespread emotional coping behaviour within this group, rather than an isolated phenomenon. On the other hand, the strengthening of emotional attachment stems from Generation Z’s identity-building as digital natives. Their youth, friendships, and even value formation have largely taken place within digital spaces. Some digital content—such as social media posts, short video clips, and chat records—is not merely piles of information but serves as archives of memory and traces of digital life, embodying growth and experiences in digital space. Revisiting these hoarded traces becomes an important way for them to affirm their existence and maintain a sense of continuous identity. Among the interviewees, 15 participants indicated that the content carries important memories or emotions, such as posts on social media, saved short videos, and chat histories. “My phone still has all the chat screenshots from my university days—those are the proof of my youth.” (M12) “If this content were lost, I would feel as if my past memories no longer existed. That’s terrifying—real life is already moving too fast, and if digital memories disappear too, then I truly become someone without a past.” (F7) These stored items possess high digital trace value (Z. Zhang & Liu, 2024), and cherishing them represents a compensatory reaction to the fleeting and unstable nature of real-world relationships.

Under emotion-driven mechanisms, Chinese Generation Z’s resistance to deletion is particularly striking. This resistance is essentially a fear of digital loss, which at a deeper level reflects the weakening of support systems in real society. In Pathway 4, perceived usefulness is absent, yet hoarding behaviour remains strongly established—indicating that even when content has no clear current utility, users still tend to retain it. As one participant explained, “I know full well that some of this content is no longer useful, but I just can’t delete it. It feels like removing it would erase part of my online life from the past few years.” (M10) This behavioural logic represents a typical emotional-locking response: once psychological attachment is formed, the items are perceived as scarce resources that are difficult to part with. In addition, in this pathway, media dependence and perceived ease of use are absent or marginalised, indicating that Chinese Generation Z’s digital behaviours have strong internal drives. They are not simply “captured” by platform design but rather actively choose certain platforms as digital homes for emotional sustenance. Even if the user experience is poor, hoarding behaviour remains strong. Twelve male and ten female participants referred to their digital space as “my little world,” “my space,” or “my secret base,” suggesting that the digital realm provides them with an emotional sanctuary of their own. “One platform I use is actually too cluttered, but most of the anime content I love is there. It feels like my spiritual corner, so I keep using it and save a lot of my favorite stuff.” (M9) “I can be myself in this little world—no one here knows me, and I don’t have to care about how others see me. Everything I browse and save is what I truly like. It makes me feel relaxed and safe.” (F1) This reflects that in the emotional-security–attachment path, media dependency manifests primarily on the emotional level. Respondents tend not to emphasise platform functionality but rather value the platform’s role as a vessel for emotional sustenance. This represents a typical emotional anchoring response, in which hoarded digital items function not merely as information but as extensions and defences of the self’s emotions.

The “Emotional-Security Attached” pathway demonstrates that the digital hoarding of Chinese Generation Z youth results from the interplay of external social pressures, internal emotional needs, and psychological defence mechanisms, forming a media behavioural pattern centred on emotional fulfilment, risk avoidance, and the maintenance of self-identity. This pattern not only underscores the emotional autonomy of Chinese Generation Z in media use during the digital–intelligent era but also reminds us that digital hoarding behaviours often conceal a deeper need for unmet emotional support and a profound yearning for stable identity in the real world.

### 5.3. Context Dependence and Multidimensional Trade-Offs: Dynamic Characteristics of Digital Hoarding Among Chinese Generation Z

The traditional TAM is rooted in instrumental rationality, attempting to depict a linear technology acceptance pathway centred on “perceived usefulness” and “perceived ease of use.” However, this study reveals that Chinese Generation Z’s digital hoarding behaviour is not a simple act of technology adoption but rather the outcome of a continuous dynamic negotiation among users and media, emotions and cognition, individual expectations and structural social pressures. Building on TAM, this study introduces additional variables—media dependency, uncertainty avoidance, emotional attachment, and deletion barriers—and applies the fsQCA method to identify two typical pathways. These findings not only uncover the multiple logics underlying Chinese Generation Z’s digital hoarding behaviours, but also map the different strategies Generation Z adopts when coping with China’s highly competitive and uncertain social environment.

The utility-integration-driven pathway centres on the idea that digital hoarding among Chinese Generation Z is not merely a method of information storage, but a proactive cognitive extension practice. Rather than being passively immersed in media structures, they actively construct self-directed knowledge management systems through digital platforms under the pressure of “self-valorisation” anxieties. In doing so, they build personal cognitive safety nets to cope with intense academic and professional competition—an intentional effort to seek control and avoid future risks within an environment of involution. This rationally driven hoarding behaviour reflects a dual psychological mechanism of “maximising efficiency” and “minimising risk”. Individuals strengthen their sense of control through hoarding; however, this pursuit of control may evolve into “efficiency anxiety” and “information overload”. In this process, media platforms are perceived as an “external brain”—an extension of the self that functions as a collaborative partner in maintaining personal competitiveness and cognitive order. In the emotional-security–attached pathway, when confronted with interpersonal alienation and fast-paced social pressures, the digital content hoarded by Chinese Generation Z is not only a collection of knowledge and resources but also an emotional testimony intertwined with memory, self-identity, and life narrative. These hoarded digital items become both a symbol of psychological security and a repository of identity stability. This behaviour functions as a form of self-healing, alleviating loneliness and countering feelings of real-world loss of control. For this generation, digital space becomes an emotional sanctuary and a “digital home” that extends their sense of personhood. The core psychological mechanism here lies in the individual’s effort to preserve digital content as a means of extending self-memory and resisting feelings of loss of control. At this stage, media platforms become emotionalised—their functions transcend instrumental use and evolve into spaces for emotional containment and the continuity of selfhood. To illustrate the distinctions and underlying logic of the two configurations more clearly, this study presents a systematic comparison between the utility-integration-driven pathway and the emotional-security–attached pathway, as shown in Table 6.

Although these two pathways lie at opposite poles of rationality and emotion, they are far from mutually exclusive. In practice, most young people’s behavioural patterns shift and recombine under the interaction of situational pressures, media characteristics, and individual cognition. By employing fsQCA, this study not only identifies the differentiations across pathways but also uncovers the diversity of behavioural logics within the group and the legitimacy of individual differences. Such configurational thinking breaks through the stable assumptions of traditional models regarding variable effects, demonstrating that Chinese Generation Z’s digital hoarding behaviour results from multiple conditions being activated and combined under specific social pressure contexts.

The complexity of Chinese Generation Z’s media behaviour configurations is closely tied to their “born-digital” upbringing, but the fundamental driving force arises from their reality as bearers of societal pressure. Growing up entirely in the post-internet era, their media habits, cognitive preferences, and psychological structures carry deep imprints of the media ecology. They are both technological natives and emotional digital migrants—adept at using media to manage real-world anxieties, fill emotional gaps, and construct digital order through system convenience and algorithmic guidance. Thus, the digital hoarding behaviour of Chinese Generation Z represents a complex coupling between external social pressures and internal psychological needs, mediated by digital technologies. It is simultaneously a technological phenomenon, a social phenomenon, and a psychological phenomenon. Therefore, the digital hoarding of this generation should not merely be understood within an instrumentalist framework but must be interpreted through a broader sociological perspective—integrating their media ecological adaptability, emotional–psychological responses, and identity–practice dimensions.

## 6. Conclusions

### 6.1. Theoretical Implications

This study is grounded in the TAM, yet ultimately transcends its rationalist paradigm. The traditional TAM assumes that technology acceptance behaviour is linearly driven by variables such as “perceived usefulness” and “perceived ease of use”, emphasising a unidirectional relationship among variables. By introducing key dimensions such as media dependency, uncertainty avoidance, emotional attachment, and deletion barriers and employing the fsQCA method, it uncovers the multiple configurational pathways underlying Chinese Generation Z’s digital hoarding behaviours. The fsQCA results indicate that digital hoarding behaviour can be achieved through multiple configurational pathways, with different combinations of conditional variables leading to high-consistency outcomes. This suggests that digital hoarding is not linearly driven by a single factor but rather results from contextual, interactive, and nonlinear causal mechanisms. At the theoretical level, this study makes a twofold contribution. On the one hand, it extends the application boundaries of the traditional TAM, shifting the paradigm from “technology adoption” to “social adaptation.” It systematically reveals the coupling of functional and emotional motivations in Chinese Generation Z’s digital hoarding, thereby deepening the understanding of the cognitive logics and psychological mechanisms behind their media practices. It deepens the understanding of the concurrent causality and contextual dependence underlying media use behaviours among Chinese Generation Z. On the other hand, the study systematically integrates variables such as emotional attachment and deletion barriers—factors that have rarely been incorporated into the TAM framework—into the analysis. By doing so, it broadens the dimensional scope and explanatory paradigms of digital media behaviour research, promotes the systematic inclusion of emotional factors in the study of Chinese youth’s digital behaviours, and further strengthens the theoretical foundation of such research. Furthermore, this research provides a theoretical framework for understanding how digital technologies become deeply embedded in and reshape contemporary youth’s social survival strategies and psychological adaptation mechanisms. It successfully redirects the focus of digital behaviour research from “how people accept technology” to “how people use technology to cope with society.”

### 6.2. Practical Implications

At the practical level, the findings of this study offer insights for platform designers, educators, and policymakers worldwide in building a healthier and more human-centred digital social ecosystem. This research calls for a more socially responsible approach to technological design: platforms should not endlessly pursue user retention time and content accumulation but instead recognise that their products have become crucial environments through which users cope with real-world pressures. For utility-integration-driven users, platforms can strengthen instrumental features such as intelligent categorisation and collection reminders to improve storage efficiency and enhance users’ sense of control over information—thereby meeting their core need for the efficient and orderly management of digital content. For emotional-security–attached users, platforms should attend to their needs for emotional maintenance and identity continuity by designing features such as emotional tagging, memory retrospectives, and soft-delete mechanisms, which can reduce resistance, anxiety, and feelings of loss during deletion. Chinese Generation Z users are deeply embedded in platform use practices, and their digital hoarding behaviours reflect a process of dynamic adjustment between information overload and psychological security needs. Platforms should recognise and respect the psychological tension between “digital nostalgia” and “information decluttering,” encouraging users to find a middle ground between digital hoarding and digital minimalism that aligns with their own media habits, thereby promoting a more sustainable digital lifestyle. Moreover, the digital hoarding behaviours of Chinese Generation Z serve as a window into the mental health conditions of contemporary youth. Educational practice should go beyond training in digital literacy skills and instead focus on cultivating digital psychological resilience. This involves guiding young people to identify the social pressures and emotional motivations behind their behaviours, nurturing critical awareness of their coexistence with digital technologies, and enhancing their self-regulation capacity to mitigate the negative consequences of excessive hoarding. Ultimately, this study demonstrates that youth digital behaviour issues are reflections of broader social problems. To fundamentally promote healthy digital lifestyles, joint efforts across society are required to alleviate the pervasive anxieties faced by young people in education, employment, and social interactions, and to construct a more supportive real-world environment.

### 6.3. Limitations and Future Directions

This study offers a valuable exploration in theoretical construction and behavioural path identification; however, certain limitations remain, warranting further expansion and refinement in future research. First, in terms of sample scope, although 35 representative Chinese Generation Z participants were selected—sufficient to meet the heterogeneity requirements of fsQCA—the sample size remains relatively limited. Future studies could extend to larger and more regionally diverse populations to enhance the generalizability and robustness of the conclusions. Second, at the methodological level, this study primarily relies on cross-sectional data and has not incorporated the temporal dynamics of digital hoarding behaviours. Future studies could combine longitudinal interviews and behavioural tracking to explore how media practices evolve dynamically with individual life cycles and broader macro-social changes. Finally, this study did not systematically compare Generation Z with other generational cohorts. Future research could include Millennials and other age groups for comparative analysis, in order to examine how varying social stressors shape distinct digital adaptation strategies across generations, thereby offering deeper insights into the interplay between technological behaviours and social structures.

## Figures and Tables

**Table 1:** Demographic Information of Participants.

No.	ID	Gender	Age	Occupation	Interview Date	Interview Duration (min)
1	F1	Female	29	Primary School Teacher	2025.3	52
2	M1	Male	28	Hotel Manager	2025.3	68
3	F2	Female	23	Student from a master’s program in Chinese Language and Literature	2025.3/2025.7	74/42
4	M2	Male	24	Student from a master’s program in Law	2025.3	58
5	M3	Male	22	Student from a master’s program in Medicine	2025.3	95
6	F3	Female	26	Brand Designer	2025.3/2025.7	49/38
7	F4	Female	23	Student from a master’s program in Journalism	2025.3	71
8	F5	Female	26	Marketing Professional	2025.3	55
9	F6	Female	24	Freelancer	2025.3	65
10	M4	Male	18	Undergraduate Student	2025.4/2025.7	79/35
11	M5	Male	22	Student from a master’s program in Communication Studies	2025.4	51
12	F7	Female	29	University Lecturer	2025.4	67
13	M6	Male	25	Wedding Host	2025.4	76
14	M7	Male	24	Student from a master’s program in Chemistry	2025.4	80
15	F8	Female	28	University Lecturer	2025.4	61
16	M8	Male	26	High School Teacher	2025.4/2025.7	73/40
17	M9	Male	23	Military Personnel	2025.4	85
18	F9	Female	17	Undergraduate student in Computer Science	2025.4	54
19	M10	Male	26	Self-employed	2025.4	66
20	M11	Male	28	Television Host	2025.4	78
21	F10	Female	25	Government Staff	2025.4	50
22	M12	Male	26	Government Staff	2025.5/2025.7	69/38
23	F11	Female	22	Online Streamer	2025.5	75
24	M13	Male	24	Marketing Manager	2025.5	59
25	F12	Female	16	High School Student	2025.5	46
26	M14	Male	26	Physician	2025.5/2025.7	63/45
27	M15	Male	28	Salesperson	2025.5	70
28	M16	Male	15	High School Student	2025.5/2025.7	56/30
29	F13	Female	24	Primary School Teacher	2025.5	64
30	F14	Female	16	High School Student	2025.5	90
31	F15	Female	25	Middle School Teacher	2025.5	48
32	M17	Male	17	Undergraduate student in Physical Education	2025.5	68
33	M18	Male	15	High School Student	2025.5	53
34	F16	Female	27	Art Teacher	2025.5/2025.7	60/47
35	F17	Female	28	Accountant	2025.5	54

**Table 2:** Calibration Anchor Points Selection Table.

Variable		Full Membership	Crossover Point	Full Non-Membership
Outcome Variable	Acceptance Effect	5	3.5	2.05
Condition Variables	Media Dependency	5	3.67	1.9
	Perceived Usefulness	5	4.5	2.2
	Perceived Ease of Use	5	4.5	2.85
	Uncertainty Avoidance	4.65	4	2.35
	Emotional Attachment	5	3.5	1.5
	Deletion Barriers	5	4.5	2.2

**Table 3:** Results of Single-Factor Necessity Analysis.

Condition Variables	Consistency	Coverage
Media Dependency	0.749376	0.753465
~Media Dependency	0.576142	0.636680
Perceived Usefulness	0.791599	0.775562
~Perceived Usefulness	0.490502	0.558142
Perceived Ease of Use	0.724303	0.744090
~Perceived Ease of Use	0.503582	0.543776
Uncertainty Avoidance	0.722674	0.848802
~Uncertainty Avoidance	0.621350	0.592844
Emotional Attachment	0.712363	0.722599
~Emotional Attachment	0.588842	0.644491
Deletion Barriers	0.585423	0.700454
~Deletion Barriers	0.706502	0.664184

Note: “~” denotes the logical operation “NOT,” meaning the condition variable does not exist.

**Table 4:** Configurational Path Analysis Results of Acceptance Attitudes toward Digital Hoarding Behaviour among Chinese Generation Z.

Condition Variables	H1	H2	H3	H4	H5	H6
Media Dependency	●	⊗	⊗	⊗	⊗	●
Perceived Usefulness	●	●	●	⊗	●	●
Perceived Ease of Use		⊗	●	●	●	●
Uncertainty Avoidance	●	●	⊗	●	●	●
Emotional Attachment	⊗	●	⊗	●	⊗	●
Deletion Barriers	⊗	●	⊗	⊗	●	
Raw Coverage	0.305547	0.204114	0.247531	0.222566	0.25616	0.486432
Unique Coverage	0.0261588	0.015793	0.00010854	0.0228482	0.032617	0.186421
Consistency	0.960751	0.984297	0.912018	0.969274	0.911373	0.949068
Solution Coverage	0.626777					
Solution Consistency	0.920312					

Note: ● = Core condition present, ● = Marginal condition present; ⊗ = Core condition absent, ⊗ = Marginal condition absent.

**Table 5:** Characteristics of Typical Configurational Paths for Acceptance Attitudes toward Digital Hoarding Behaviour among Chinese Generation Z.

Configurational Type	Corresponding Configurations	Configurational Characteristics	Configurational Implications
Utility-Integration-Driven	H1, H3, H5, H6	Focus on function orientation, instrumental rationality, and efficiency optimisation.	Hoarding behaviour manifests as a rational and strategic form of retention, emphasising content usefulness and platform operation convenience.
Emotional-Security–Attached	H2, H4	Focus on emotional support, risk avoidance, and psychological comfort.	Hoarding behaviour reflects a coping response that seeks reassurance through retention, highlighting the emotional value of content and a sense of future security.

**Table 6:** Comparison of the Two Configurational Pathways of Digital Hoarding Behaviour Among Chinese Generation Z.

Configurational Type	Utility-Integration-Driven	Emotional-Security–Attached
Behavioural Logic	Instrumental-rationality-oriented, emphasising functional efficacy and cognitive control.	Emotionally attached–oriented, emphasising psychological comfort and emotional stability.
Psychological Mechanism	Strategic behaviour aimed at self-enhancement and risk avoidance.	Compensatory behaviour centred on security and emotional continuity.
Media Relationship	Active integration of media functions to enhance efficiency.	Passive dependence on media for emotional solace.
Social Roots	involutional competition, performance anxiety, and achievement pressure.	Interpersonal alienation, emotional isolation, and a sense of identity drift.

## Data Availability

The original contributions presented in this study are included in the article. Further inquiries can be directed to the corresponding author.
